# Association of *ABCB1* Genetic Variants with Epilepsy Susceptibility in Jordanian Cohort

**DOI:** 10.3390/neurolint18050075

**Published:** 2026-04-22

**Authors:** Rami Abduljabbar, Al-Motassem Yousef, Duaa Eid Tamimi, Shayma Z. Abdullah, Zhenbao Liu

**Affiliations:** 1 Department of Biopharmaceutics and Clinical Pharmacy, School of Pharmacy, The University of Jordan, Amman 11942, Jordan; ramiabduljabbar@csu.edu.cn (R.A.); s.abdullah@ju.edu.jo (S.Z.A.); 2 Department of Pharmaceutics, Xiangya School of Pharmaceutical Sciences, Central South University, Changsha 410017, China; zhenbaoliu@csu.edu.cn; 3 Department of Pharmacology, School of Medicine, The University of Jordan, Amman 11942, Jordan; do3a2tamimi@yahoo.com

**Keywords:** epilepsy, *ABCB1*, susceptibility, alleles, genotypes, polymorphisms

## Abstract

Background: Epilepsy is a chronic disorder with a higher prevalence in low- and middle-income countries. ATP-binding cassette superfamily B1 (*ABCB1*) not only has a potential influence on the resistance to antiepileptic drugs but also plays a possible role in the occurrence of epilepsy. Purpose: To evaluate the association of *ABCB1* polymorphisms, c.1236C>T (rs1128503), c.2677G>T (rs2032582), and c.3435C>T (rs1045642), with epilepsy susceptibility in a Jordanian cohort. Subjects and methods: Eighty-six cases of patients with epilepsy were analyzed using polymerase chain reaction (PCR) for *ABCB1* c.1236C>T, c.2677G>T, and c.3435C>T gene variants. The proportions of genotypes and alleles in the epilepsy group were compared with one hundred healthy controls who were previously also analyzed by PCR. Results: The C alleles of the *ABCB1* polymorphisms c.1236C>T and c.3435C>T were more prevalent in the epilepsy group than in controls. The patients with epilepsy were less likely to have the TT genotype compared with controls (concerning *ABCB1* c.1236C>T) (OR_TT vs. CC_ = 0.42; 95% CI = [0.19–0.91]; *p* = 0.019). The CC genotype of *ABCB1* c.3435C>T was more frequent in epileptics than healthy people (OR_CC vs. TT_ = 4.3; 95% CI = [1.8–9.95]; *p* = 0.0007). No significant difference in *ABCB1* c.2677G>T allelic and genotypic frequencies was observed between epileptic cases and healthy volunteers. Conclusion: Our findings suggest that *ABCB1* c.1236C>T and c.3435C>T variants were associated with epilepsy susceptibility in this Jordanian cohort, whereas no significant association was observed for c.2677G>T. These findings should be interpreted cautiously because of the modest sample size and require validation in larger, independent studies.

## 1. Introduction

Epilepsy is characterized by unprovoked seizures, affects over 70 million people worldwide [1,2,3] and is associated with low quality of life and comorbidities [4]. The prevalence and incidence of epilepsies are higher in countries, including Jordan, with low and middle income, totaling 80% of global epilepsy, compared with high-income countries [5]. The etiology of epilepsy is complex and not yet fully understood; however, genetic mutations, such as single-nucleotide polymorphisms (SNPs), are known contributors to epilepsy susceptibility [6,7].

The efflux transporter proteins like P-glycoprotein (P-gp) in the blood–brain barrier may influence resistance to antiepileptic drugs (AEDs) and play a role in epilepsy occurrence [8]. The multidrug resistance 1 transporter, named ATP-binding cassette superfamily B1 (*ABCB1*) transporter, was identified to contribute to resistance to AEDs [8,9]. The *ABCB1* gene spans chromosome 7 and consists of 28 exons [10]. More than 50 SNPs have been reported in this gene, and three of them are more frequent [c.1236C>T (rs1128503), c.2677G>T (rs2032582), and c.3435C>T (rs1045642)] [11]. Two SNPs, c.1236C>T (Gly412Gly) and c.3435C>T (Ile1145Ile), are synonymous, while c.2677G>T (Ala893Ser/Thr) is a non-synonymous polymorphism [11,12]. Many studies have found that these three SNPs are associated with the development of numerous diseases, including epilepsy, with controversial results [13]. Because *ABCB1* has been implicated in both antiepileptic drug response and disease susceptibility, it is important to distinguish these questions. The present study addresses epilepsy susceptibility (case–control comparison with healthy controls), not pharmacoresistance. Thus, in our current study, we evaluated the association between genetic polymorphisms of three *ABCB1* SNPs (c.1236C>T, c.2677G>T, and c.3435C>T) and the risk for epilepsy among the population in Jordan.

## 2. Materials and Methods

### 2.1. Patient Recruitment

Eligible participants were Jordanian patients with epilepsy diagnosed by neurologists based on clinical symptoms, magnetic resonance imaging, and electroencephalogram findings at the neurology clinics of Jordan University Hospital and Al-Basheer Hospital in Amman, Jordan. Participants were 18 years of age or older. Specialized neurologists diagnosed epileptic seizures following the updated criteria and categorization recommendations of the International League Against Epilepsy (ILAE). A patient is considered to have epilepsy when they have experienced two or more unprovoked seizures separated by more than 24 h and have been prescribed antiepileptic drugs for at least three months [14].

On the other hand, we excluded patients with conditions that could increase epilepsy occurrence, such as neurological or systemic degenerative disorders, tumors, atrophic lesions, tuberculoma, and multiple neurocysticercoses [15,16,17], gross neurological deficiencies, and hematopoietic [18], traumatic, metabolic, and psychiatric diseases [19]. In addition, patients with insufficient medical records were also excluded from our work. We invited one hundred patients, but eighty-six met the recruitment criteria and were included in our study. Throughout the study, the rights and confidentiality of the human subjects were maintained. The proportions of genotypes and alleles in the epilepsy group were compared with the non-epilepsy healthy control cohort that was published previously [10].

### 2.2. Sample Size Calculation

We determined the sample size using Tabachnick and Fidell’s recommendations for logistic regression analysis because there is no comparable research from Jordan to use as a guide [20]. According to their criteria, five to twenty individuals are needed for each independent variable. Our study had 6 independent variables, and we used 10 participants per variable. We determined that a sample size of at least 60 participants for each group would be enough. However, we invited one hundred patients with epilepsy to account for potential sample loss, inadequate patient data, non-cooperation, and reaction failures (PCR, and DNA extraction and sequencing).

### 2.3. DNA Extraction and ABCB1 Gene Polymorphism Analysis

A 3 mL blood sample was collected from all recruited patients, and DNA was isolated from all these samples using a standard salting-out procedure [21]. We designed the primers for polymerase chain reaction (PCR) amplification and extension using the Primer-Blast program (Table 1). PCR conditions included the following thermal cycle parameters: an initial denaturation step at 94 °C for three minutes, followed by 39 cycles of denaturation at 94 °C for half a minute, annealing at 62 °C for fifteen seconds, an extension step at 68 °C for one minute, and a final extension step at 68 °C for five minutes. This PCR program was used for the three studied SNPs. The sequencing was done for all sharp PCR products using the Sanger technique by GENEWIZ Technical Support Group, South Plainfield, NJ, USA (http://www.genewiz.com). Also, the same genotyping method, PCR, was used for the healthy controls [10].

### 2.4. Statistical Analysis

All statistical analyses were performed using SPSS software version 22 (SPSS^®^ Inc., Chicago, IL, USA). Categorical variables of baseline characteristics were presented as frequencies and proportions. We used Chromas Lite software version 2.1.1 to read the results of the sequencing and alleles. Then, genotype and allele frequencies among participants were calculated. Differences in *ABCB1* polymorphisms were assessed using chi-square or Fisher’s exact, as appropriate. The chi-square test was used to assess the concordance of the frequencies of genotypes and alleles with the Hardy–Weinberg equilibrium (degree of freedom = 1). The odds ratio (OR) was calculated with 95% confidence intervals (CIs). Statistical significance was defined as two-tailed at *p* < 0.05. Linkage disequilibrium (LD) was calculated by the Multiallelic Interallelic Disequilibrium Analysis Software (MIDAS) and then measured by Lewontin’s coefficient (D’) and rHo square (r^2^) values [22].

## 3. Results

**Figure 1:**
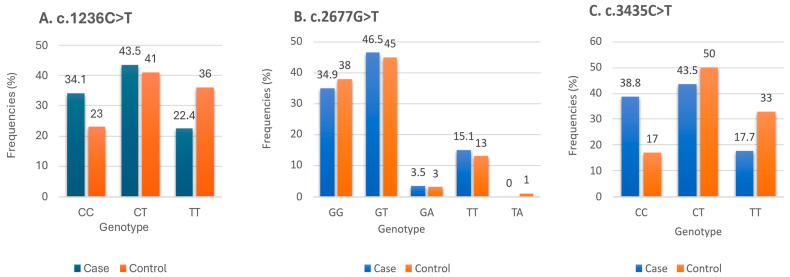
Genotype frequencies among case and control groups. (**A**) Genotype frequencies in c.1236C>T SNP. (**B**) Genotype frequencies in c.2677G>T SNP. (**C**) Genotype frequencies in c.3435C>T SNP.

**Figure 2:**
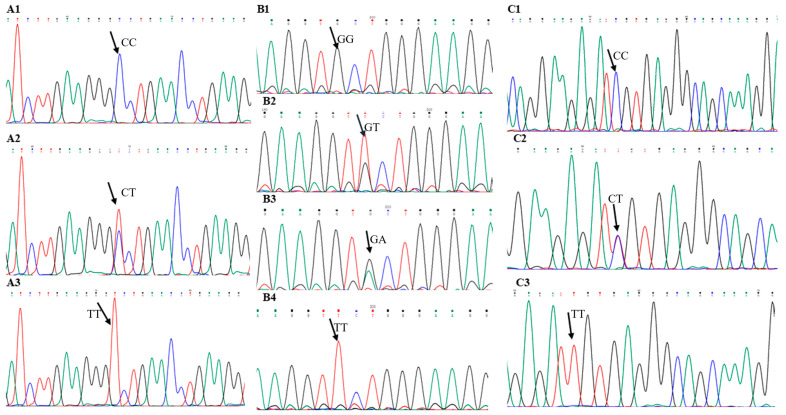
*ABCB1* variants in Jordanian patients with epilepsy. (**A1**) c.1236C>T homozygous CC genotype. (**A2**) c.1236C>T heterozygous CT genotype. (**A3**) c.1236C>T homozygous TT genotype. (**B1**) c.2677G>T homozygous GG genotype. (**B2**) c.2677G>T heterozygous GT genotype. (**B3**) c.2677G>T heterozygous GA genotype. (**B4**) c.2677G>T homozygous TT genotype. (**C1**) c.3435C>T homozygous CC genotype. (**C2**) c.3435C>T heterozygous CT genotype. (**C3**) c.3435C>T homozygous TT genotype.

Table 2 summarizes the demographic and clinical characteristics of the study participants. All studied *ABCB1* polymorphisms (c.1236C>T, c.2677G>T, and c.3435C>T) were in Hardy–Weinberg equilibrium (*p* > 0.05). The distributions of genotype and allele proportions of *ABCB1* SNPs of patients with epilepsy and healthy controls are shown in Table 3. When we compared the *ABCB1* variants of the epilepsy cohort with healthy individuals, we observed statistically significant differences with two SNPs, c.1236C>T (rs1128503) and c.3435C>T (rs1045642), at the levels of genotypes and alleles. For c.1236C>T (rs1128503), the results revealed that the patients with epilepsy were less likely to have TT genotypes than CC (OR_TT vs. CC_ = 0.42; 95% CI = [0.19–0.91]; *p* = 0.019) and CT genotypes as compared with controls (OR_TT vs. others_ = 0.5; 95% CI = [0.2–0.9]; *p* = 0.03). In addition, the cases with the C allele were two times more susceptible to developing epilepsy than those with the T allele (OR = 2.07; 95% CI = [1.2–3.6]; *p* = 0.01). Regarding c.3435C>T (rs1045642), we found the CC genotype was more frequent in epileptics than healthy people (OR_CC vs. TT_ = 4.3; 95% CI = [1.8–9.95]; *p* = 0.0007) (OR_CC vs. others_ = 3.1; 95% CI = [1.6–6]; *p* = 0.0007). On the contrary, the TT genotype was less frequent in cases than in controls (OR = 0.4; 95% CI = [0.2–0.8]; *p* = 0.01). No significant difference in c.2677G>T (rs2032582) allelic and genotypic frequencies was observed between epileptic cases and healthy volunteers. Figure 1 shows the distributions of genotype percentages for *ABCB1* SNPs in patients with epilepsy and healthy controls. Figure 2 shows the sequencing results of the three studied *ABCB1* variants among Jordanian patients with epilepsy.

Further analysis based on dominant and recessive models of the *ABCB1* c.3435C>T polymorphism revealed that patients with epilepsy were more likely to have at least one C allele (OR = 2.3; 95% CI = [1.1–4.6]; *p* = 0.02). In contrast, healthy individuals were less likely to have at least one T allele compared to patients (OR = 0.3; 95% CI = [0.2–0.6]; *p* = 0.001). Additionally, an analysis of dominant and recessive models of the other two variants (c.1236C>T and c.2677G>T) did not reveal statistical significance. Regarding haplotypes, the standardized, pair-wise LD value was calculated for each pair of markers. All pairs showed strong LD among the patient group. The loci c.3435C>T - c.2677G>T, c.3435C>T - c.1236C>T, and c.2677G>T - c.1236C>T exhibited (D’ = 0.86, r^2^ = 0.718; D’ = 0.823, r^2^ = 0.558; and D’ = 0.991, r^2^ = 0.833) respectively. When comparing these LD values with those previously published for healthy Jordanian individuals, only one pair of markers (c.3435C>T - c.2677G>T) showed slightly strong LD (D’ = 0.765; r^2^ = 0.426). The other two pairs (c.3435C>T - c.1236C>T and c.2677G>T - c.1236C>T) demonstrated weaker LD (D’ = 0.319, r^2^ = 0.091; and D’ = 0.303, r^2^ = 0.087 respectively) [11].

## 4. Discussion

Epilepsy is a chronic neurological disorder affecting 1% of the population worldwide, 40% of whom have a form of epilepsy related to genetic causes [23]. The human *ABCB1* gene encodes P-gp, which is a member of the efflux transporters and has a role in the pharmacoresistance of epilepsy [24,25]. Recently, it has been suggested that *ABCB1* polymorphisms may be associated with epilepsy susceptibility [26,27]. Several studies have focused on the potential role of the *ABCB1* gene in drug resistance to antiepileptic drugs [27,28,29,30,31] and a more recent one was in Jordan [12]. The current study aimed to evaluate whether certain polymorphic genotypes and alleles of the *ABCB1* SNPs (c.1236C>T, c.2677G>T, and c.3435C>T) are more prevalent in Jordanian patients with epilepsy versus healthy subjects. The literature on *ABCB1* and epilepsy-related phenotypes is inconsistent. While some reports suggested an association between *ABCB1* variants and drug-resistant epilepsy, larger prospective and meta-analytic studies have been less supportive. In a prospective cohort of 503 patients, Leschziner et al. found no evidence that common ABCB1 variation influenced seizure or drug-withdrawal outcomes after initiation of antiepileptic therapy [32]. Likewise, Tan and Berkovic highlighted methodological challenges and conflicting results in the field [33]. With respect to epilepsy susceptibility, NurMohamed et al. reported in a meta-analysis of nine case–control studies that ABCB1 c.3435C>T was not associated with the overall risk of epilepsy [34]. Therefore, our results should be interpreted in the context of this heterogeneous literature and viewed as population-specific, exploratory findings rather than definitive evidence.

The results of the present study showed a strong association between *ABCB1* variants (c.1236C>T and c.3435C>T) and predisposition to epilepsy at the level of genotypes and alleles. On the contrary, our study did not find a significant association between the *ABCB1* c.2677G>T polymorphism and epilepsy susceptibility. No prior studies have investigated the effects of the c.1236C>T SNP on epilepsy occurrence. Only one previous study investigated the association between the *ABCB1* c.1236C>T variant and the risk of infantile spasms (West syndrome) which is an epilepsy syndrome among children. The results of this Han Chinese study reported no significant differences in allelic and genotypic frequencies of c.1236C>T polymorphisms between the cases with infantile spasms and controls [35]. This finding contradicts our results, which confirmed that the carriers of the C allele are more susceptible to epilepsy.

With regard to the *ABCB1* c.2677G>T SNP, two previous studies verified the effect of the c.2677G>T polymorphism on epilepsy vulnerability. The first study was conducted among Han Chinese people and its results were consistent with our findings [35]. Dong et al. revealed the frequency of *ABCB1* c.2677G>T genotypes did not differ significantly between the two study groups, infantile spasm cases and healthy volunteers [35]. The second one was done among the Caucasian population and showed that G allele carriers in young epileptics were more likely to develop epilepsy compared with healthy participants [36].

Regarding c.3435C>T, previous studies revealed conflicting findings regarding the association with epilepsy occurrence. Balan et al. observed a significant association with c.3435C>T at a genotypic and allelic level. They found the TT genotype and the T allele were overrepresented in patients with epilepsy. Others also have similar findings [8,35]. Their conclusion was inconsistent with our findings, which indicated that patients with epilepsy are more likely to have CC genotypes and C alleles [8]. Additionally, Ponnala et al. revealed that the T allele of the *ABCB1* c.3435C>T SNP was higher in the Indian epileptic population compared with controls [37]. Consistent with our results, four studies revealed similar relationships between the *ABCB1* c.3435C>T variants and the development of epilepsy [27,36,38,39]. Two studies reported an overrepresentation of C alleles in the patients’ group [38,39]. Otherwise, a meta-analysis performed by Nurmohamed and colleagues identified no association between *ABCB1* c.3435C>T variants and the risk of epilepsy [34]. Differences in ethnicities and sample sizes were observed and may be responsible for the conflicting observation regarding the c.3435C>T SNP.

Our study has several limitations. First, the sample size was relatively small, which may limit the generalizability of our findings. Larger studies are needed to validate these results. Second, the most significant limitation of this study is the lack of whole-exome or whole-genome sequencing. Consequently, we cannot completely exclude the presence of additional pathogenic variants in other genes that could influence the phenotype or act as modifiers. Therefore, the results should be viewed with this limitation in mind, and further research using comprehensive genomic screening is needed to confirm our findings. Third, the study population was specific to Jordanians, and the genetic variations observed may not apply to other ethnic groups. Fourth, we studied a limited number of genetic polymorphisms in the *ABCB1* gene, potentially overlooking other genetic variations. Additionally, our study did not control for all possible environmental and lifestyle factors, which could confound the association observed. Moreover, it is also critical to recognize that a significant percentage (38.4%) of our cohort’s epilepsy type could not be definitively categorized due to limited accompanying documentation, and the patients did not know about the type of epilepsy. Furthermore, although a disparity was observed in the proportion of patients with and without a family history of epilepsy (13% versus 73%), we confirmed that the association between the *ABCB1* variant and epilepsy risk, the primary finding of this study, was evident across the entire study population. To validate these results and further elucidate the potential role of family history as an effect modifier, additional research involving larger, well-powered cohorts matched for family history is warranted.

Importantly, this case–control study was not designed to evaluate pharmacoresistance, nor to determine functional consequences of the observed variants on *P*-glycoprotein expression or activity. In addition, we did not assess other genomic factors, inflammatory markers, gut-related phenotypes, or environmental confounders that may modify epilepsy risk. Our study found a significant difference in genotypic and allelic frequencies between patients with epilepsy and a healthy control group. However, a limitation of our research is the lack of matching for age and gender between these groups. This is due to our use of data from previously published healthy controls, which lacked detailed demographic information. While we acknowledge that controlling for these variables would have strengthened our findings, it is generally understood that genetic makeup is not strongly dependent on or significantly affected by age and gender. Future studies should aim to replicate our results using a control group more closely matched to our patient population to further validate our conclusions.

Taken together, these results should be regarded as exploratory and hypothesis-generating rather than as definitive evidence of a causal role for *ABCB1* variants in epilepsy susceptibility. Building on the findings of this study, several future research directions are proposed to further elucidate the role of *ABCB1*. Larger-scale studies involving broader cohorts from Jordan and other Middle Eastern populations are needed. Comparative studies between different ethnic groups would identify population-specific genetic markers. Expanding genetic analysis to include other polymorphisms within *ABCB1* and other genes could be valuable. Functional studies should investigate the biological mechanisms by which these polymorphisms influence epilepsy susceptibility. An additional area warranting future investigation is the interaction between *ABCB1* variation and inflammatory pathways, including gut–brain mechanisms. Neuroinflammation has been increasingly implicated in seizure generation and epileptogenesis, and *ABCB1*/*P*-glycoprotein has also been studied in inflammatory bowel disease in relation to epithelial barrier function, disease susceptibility, and treatment response [40,41,42]. Because our study did not include inflammatory biomarkers, gastrointestinal phenotyping, or microbiome data, we could not evaluate these hypotheses directly. Future studies integrating genetic, inflammatory, and clinical phenotypes may help clarify whether *ABCB1* links epilepsy susceptibility to systemic or intestinal inflammatory pathways. Moreover, future studies should aim to include more detailed electroclinical and neuroimaging-based phenotyping, including seizure classification and involved brain regions, to better define genotype–phenotype relationships.

## 5. Conclusions

In this Jordanian cohort, *ABCB1* c.1236C>T and c.3435C>T showed associations with epilepsy susceptibility, whereas c.2677G>T did not. Given the modest sample size, the use of previously published controls, and the inconsistent international literature, these findings should be considered preliminary and require replication in larger, well-characterized populations and functional studies.

## Figures and Tables

**Table 1:** Primer sequences for *ABCB1* SNPs.

SNPs (rs)	Primer Sequence	Exon	Amplicon Size	Tm (C°)	CG%	Self-Complementarity
c.1236C>T (rs1128503)	F: 5′- CCAGTTGATACTGCTAGAGCTT −3′ 87,599,691---712	12th	647	57.60	45.45	2.00
	R: 5′-CCTGTGTCTGTGAATTGCCTT −3′ 87,503,345----325			58.77	47.62	0.00
c.2677G>T (rs2032582)	F: 5′- AGGAGGAAAGTGGGGAGGAA −3′ 87,531,019----038	21st	480	59.80	55	0.00
	R: 5′- ATAGGTTCCAGGCTTGCTGT −3′ 87,531,494---475			59.00	50	0.00
c.3435C>T (rs1045642)	F: 5′- CTCACAAGGAGGGTCAGGTG −3′ 87,509,074----093	26th	337	59.68	60	2.00
	R: 5′- GGAGCCCATCCTGTTTGACT −3′ 87,509,410---391			59.67	55	2.00

Tm, melting temperature as calculated by Primer-BLAST software program (https://www.ncbi.nlm.nih.gov/tools/primer-blast/ (accessed on 5 April 2026)); F, forward; R, reverse.

**Table 2:** Demographic and clinical characteristics of epileptic individuals included in the study.

Subject Variable	Mean (±SD) or *n* (%) (N = 86)
Male	46 (53.5)
Female	40 (46.5)
Age at interview (years)	30.4 (±12.6)
Age at the first unprovoked seizure (years)	19.9 (±12.9)
Duration of epilepsy (years)	10.6 (±9.9)
Epilepsy type	
Generalized tonic–clonic	29 (33.7)
Partial/focal	24 (27.9)
Unknown	33 (38.4)
Epilepsy etiology	
Unknown	57 (66.3)
Trauma	16 (18.6)
Fever	5 (5.8)
Infection	4 (4.65)
Stroke	4 (4.65)
Family history	
Yes	13 (15.1)
No	73 (84.9)

Values expressed as N and %; N: number; % were calculated out of available data. SD: standard deviation.

**Table 3:** Distributions of genotypes and alleles of *ABCB1* polymorphisms.

SNP	Genotypes & Alleles	Case Frequency *n* (%) N = 86	Control Frequency *n* (%) N = 100	OR	95% CI	*p*-Value
c.1236C>T (rs1128503)	CC	29 (34.1)	23 (23)	1.7	0.9–3.3	0.08 *
	CT	37 (43.5)	41 (41)	1.1	0.6–1.9	
	TT	19 (22.4)	36 (36)	0.5	0.26–0.97	
	CC + CT	66 (77.6)	64 (64)	1.9	1.06–3.7	0.9
	TT	19 (22.4)	36 (36)			
	CT + TT	56 (65.9)	77 (77)	0.6	0.3–1.08	0.09
	CC	29 (34.1)	23 (23)			
	C	95 (55.9)	87 (43.5)	1.6	1.1–2.5	0.02
	T	75 (44.1)	113 (56.5)			
c.2677G>T (rs2032582)	GG	30 (34.9)	38 (38)	0.8	0.5–1.5	0.9 *
	GT	40 (46.5)	45 (45)	1.08	0.6–1.8	
	GA	3 (3.5)	3 (3)	1.3	0.2–6	
	TT	13 (15.1)	13 (13)	1.1	0.5–2.6	
	TA	0	1 (1)	0.3	0.01–8.1	
	AA	0	0 (0)	1	0.01–50.8	
	GG + GT + GA	73 (84.9)	86 (86)	0.9	0.4–2.02	0.8
	TT + TA	13 (15.1)	14 (14)			
	GT + TT + TA	53 (61.6)	59 (59)	1.1	0.6–1.9	0.7
	GG + GA	33 (38.4)	41 (41)			
	G	103 (59.9)	124 (62)	0.9	0.5–1.6	0.9 *
	T	66 (38.4)	72 (36)	1.08	0.6–1.9	
	A	03 (1.7)	04 (2)	0.85	0.1–7.2	
c.3435C>T (rs1045642)	CC	33 (38.8)	17 (17)	3.1	1.6–6.1	0.0004 *
	CT	37 (43.5)	50 (50)	0.77	0.43–1.38	
	TT	15 (17.7)	33 (33)	0.44	0.22–0.87	
	CC + CT	70 (82.3)	67 (67)	2.3	1.1–4.6	0.02
	TT	15 (17.7)	33 (33)	0.4	0.2–0.87	
	CT + TT	52 (61.2)	83 (83)	0.3	0.2–0.6	0.001
	CC	33 (38.8)	17 (17)	3.1	1.5–6.1	
	C	103 (60.6)	84 (42)	2.1	1.4–3.2	0.0008
	T	67 (39.4)	116 (58)	0.47	0.31–0.71	
Reference		The current study	[10]			

Fisher’s exact test or chi-square test (as appropriate) was used to estimate *p*-value; *p*, probability. A *p*-value of <0.05 is considered significant. Values expressed as %; % was calculated out of available data. *: *p*-value was calculated for all groups together using the chi-square test.

## Data Availability

Sequence data supporting the findings of this study have been deposited in the ClinVar database under accession numbers SCV006555102 —SCV006555104.

## References

[1] Hauser W.A. (2019). An unparalleled assessment of the global burden of epilepsy. Lancet Neurol..

[2] Löscher W., Potschka H., Sisodiya S.M., Vezzani A. (2020). Drug resistance in epilepsy: Clinical impact, potential mechanisms, and new innovative treatment options. Pharmacol. Rev..

[3] Ding D., Zhou D., Sander J.W., Wang W., Li S., Hong Z. (2021). Epilepsy in China: Major progress in the past two decades. Lancet Neurol..

[4] Thijs R.D., Surges R., O’Brien T.J., Sander J.W. (2019). Epilepsy in adults. Lancet.

[5] Espinosa-Jovel C., Toledano R., Aledo-Serrano Á., García-Morales I., Gil-Nagel A. (2018). Epidemiological profile of epilepsy in low income populations. Seizure.

[6] Rees M.I. (2010). The genetics of epilepsy—The past, the present and future. Seizure.

[7] Pitkänen A., Lukasiuk K. (2011). Mechanisms of epileptogenesis and potential treatment targets. Lancet Neurol..

[8] Balan S., Bharathan S.P., Vellichiramal N.N., Sathyan S., Joseph V., Radhakrishnan K., Banerjee M. (2014). Genetic association analysis of ATP binding cassette protein family reveals a novel association of *ABCB1* genetic variants with epilepsy risk, but not with drug-resistance. PLoS ONE.

[9] Ambudkar S.V., Kimchi-Sarfaty C., Sauna Z.E., Gottesman M.M. (2003). P-glycoprotein: From genomics to mechanism. Oncogene.

[10] Khabour O., Alzoubi K., Al-Azzam S., Mhaidat N.M. (2013). Frequency of MDR1 single nucleotide polymorphisms in a Jordanian population, including a novel variant. Genet. Mol. Res..

[11] Al-Diab O., Yousef A., Al Manassrah E., Masadeh A., Olemat M., Qosa H., Kherbash A., Bulatova N. (2015). Genotype and Haplotype Analysis of *ABCB1* at 1236, 2677 and 3435 among Jordanian Population. Trop. J. Pharm. Res..

[12] Tamimi D.E., Abduljabbar R., Yousef A.-M., Saeed R.M., Zawiah M. (2021). Association between *ABCB1* polymorphisms and response to antiepileptic drugs among Jordanian epileptic patients. Neurol. Res..

[13] McDonagh E.M., Whirl-Carrillo M., Garten Y., Altman R.B., Klein T.E. (2011). From pharmacogenomic knowledge acquisition to clinical applications: The PharmGKB as a clinical pharmacogenomic biomarker resource. Biomark. Med..

[14] Fisher R.S., Acevedo C., Arzimanoglou A., Bogacz A., Cross J.H., Elger C.E., Engel J., Forsgren L., French J.A., Glynn M. (2014). ILAE official report: A practical clinical definition of epilepsy. Epilepsia.

[15] Shaheen U., Prasad D., Sharma V., Suryaprabha T., Ahuja Y., Jyothy A., Munshi A. (2014). Significance of MDR1 gene polymorphism C3435T in predicting drug response in epilepsy. Epilepsy Res..

[16] Ma C.-L., Wu X.-Y., Zheng J., Wu Z.-Y., Hong Z., Zhong M.-K. (2014). Association of *SCN1A*, *SCN2A* and *ABCC2* gene polymorphisms with the response to antiepileptic drugs in Chinese Han patients with epilepsy. Pharmacogenomics.

[17] Grover S., Talwar P., Gourie-Devi M., Gupta M., Bala K., Sharma S., Baghel R., Kaur H., Sharma A., Kukreti R. (2010). Genetic polymorphisms in sex hormone metabolizing genes and drug response in women with epilepsy. Pharmacogenomics.

[18] Wang P., Zhou Q., Sheng Y., Tang B., Liu Z., Zhou B. (2014). Association between two functional SNPs of SCN1A gene and efficacy of carbamazepine monotherapy for focal seizures in Chinese Han epileptic patients. Zhong Nan Da Xue Xue Bao Yi Xue Ban.

[19] Subenthiran S., Abdullah N.R., Joseph J.P., Muniandy P.K., Mok B.T., Kee C.C., Ismail Z., Mohamed Z. (2013). Linkage disequilibrium between polymorphisms of ABCB1 and ABCC2 to predict the treatment outcome of Malaysians with complex partial seizures on treatment with carbamazepine mono-therapy at the Kuala Lumpur Hospital. PLoS ONE.

[20] Tabachnick B.G., Fidell L.S., Ullman J.B. (2013). Using Multivariate Statistics.

[21] Miller S.A., Dykes D., Polesky H. (1988). A simple salting out procedure for extracting DNA from human nucleated cells. Nucleic Acids Res..

[22] Gaunt T.R., Rodriguez S., Zapata C., Day I.N. (2006). MIDAS: Software for analysis and visualisation of interallelic disequilibrium between multiallelic markers. BMC Bioinform..

[23] Das S.K., Biswas A., Roy T., Banerjee T.K., Mukherjee C.S., Raut D.K., Chaudhuri A. (2006). A random sample survey for prevalence of major neurological disorders in Kolkata. Indian J. Med. Res..

[24] Sisodiya S.M., Lin W., Harding B.N., Squier M.V., Thom M. (2002). Drug resistance in epilepsy: Expression of drug resistance proteins in common causes of refractory epilepsy. Brain.

[25] Marchi N., Hallene K.L., Kight K.M., Cucullo L., Moddel G., Bingaman W., Dini G., Vezzani A., Janigro D. (2004). Significance of MDR1 and multiple drug resistance in refractory human epileptic brain. BMC Med..

[26] Ebid A.-H.I.M., Ahmed M.M., Mohammed S.A. (2007). Therapeutic drug monitoring and clinical outcomes in epileptic Egyptian patients: A gene polymorphism perspective study. Ther. Drug Monit..

[27] Hung C.-C., Tai J.J., Lin C.-J., Lee M.-J., Liou H.-H. (2005). Complex haplotypic effects of the ABCB1 gene on epilepsy treatment response. Pharmacogenomics.

[28] Kim D.W., Kim M., Lee S.K., Kang R., Lee S.-Y. (2006). Lack of association between C3435T nucleotide MDR1 genetic polymorphism and multidrug-resistant epilepsy. Seizure.

[29] Kwan P., Baum L., Wong V., Ng P.W., Lui C.H., Sin N.C., Hui A.C., Yu E., Wong L.K. (2007). Association between *ABCB1* C3435T polymorphism and drug-resistant epilepsy in Han Chinese. Epilepsy Behav..

[30] Sills G.J., Mohanraj R., Butler E., McCrindle S., Collier L., Wilson E.A., Brodie M.J. (2005). Lack of association between the C3435T polymorphism in the human multidrug resistance (MDR1) gene and response to antiepileptic drug treatment. Epilepsia.

[31] Siddiqui A., Kerb R., Weale M.E., Brinkmann U., Smith A., Goldstein D.B., Wood N.W., Sisodiya S.M. (2003). Association of multidrug resistance in epilepsy with a polymorphism in the drug-transporter gene ABCB1. N. Engl. J. Med..

[32] Leschziner G., Jorgensen A.L., Pirmohamed M., Williamson P.R., Marson A.G., Coffey A.J., Middleditch C., Rogers J., Bentley D.R., Chadwick D.W. (2006). Clinical factors and ABCB1 polymorphisms in prediction of antiepileptic drug response: A prospective cohort study. Lancet Neurol..

[33] Tan N.C., Berkovic S.F. (2006). Prediction of drug resistance in epilepsy: Not as easy as ABC. Lancet Neurol..

[34] Nurmohamed L., Garcia-Bournissen F., Buono R.J., Shannon M.W., Finkelstein Y. (2010). Predisposition to epilepsy—Does the *ABCB1* gene play a role?. Epilepsia.

[35] Dong L., Mao M., Luo R., Tong Y., Yu D. (2011). Common ABCB1 polymorphisms associated with susceptibility to infantile spasms in the Chinese Han population. Genet. Mol. Res..

[36] Ufer M., Mosyagin I., Muhle H., Jacobsen T., Haenisch S., Häsler R., Faltraco F., Remmler C., von Spiczak S., Kroemer H.K. (2009). Non-response to antiepileptic pharmacotherapy is associated with the ABCC2− 24C>T polymorphism in young and adult patients with epilepsy. Pharmacogenetics Genom..

[37] Ponnala S., Chaudhari J.R., Jaleel M.A., Bhiladvala D., Kaipa P.R., Das U.N., Hasan Q. (2012). Role of MDR1 C3435T and GABRG2 C588T gene polymorphisms in seizure occurrence and MDR1 effect on anti-epileptic drug (phenytoin) absorption. Genet. Test. Mol. Biomark..

[38] Grover S., Bala K., Sharma S., Gourie-Devi M., Baghel R., Kaur H., Gupta M., Talwar P., Kukreti R. (2010). Absence of a general association between ABCB1 genetic variants and response to antiepileptic drugs in epilepsy patients. Biochimie.

[39] Tang K., Ngoi S.-M., Gwee P.-C., Chua J.M.Z., Lee E.J.D., Chong S.S., Lee C.G.L. (2002). Distinct haplotype profiles and strong linkage disequilibrium at the MDR1 multidrug transporter gene locus in three ethnic Asian populations. Pharmacogenetics Genom..

[40] Vezzani A., French J., Bartfai T., Baram T.Z. (2011). The role of inflammation in epilepsy. Nat. Rev. Neurol..

[41] Vezzani A., Friedman A., Dingledine R.J. (2013). The role of inflammation in epileptogenesis. Neuropharmacology.

[42] Petryszyn P.W., Wiela-Hojeńska A. (2018). The importance of the polymorphisms of the ABCB1 gene in disease susceptibility, behavior and response to treatment in inflammatory bowel disease: A literature review. Adv. Clin. Exp. Med..

